# Supplementary material for: Expression and Immune Response Profiles in Nile Tilapia (Oreochromis niloticus) and European Sea Bass (Dicentrarchus labrax) During Pathogen Challenge and Infection
Source: Int J Mol Sci. 2024 Nov 28;25(23):12829. doi: 10.3390/ijms252312829 (PMC11641086; doi:10.3390/ijms252312829)
Supplement: Supplementary file 1 [file ijms-25-12829-s001.zip › ijms-3341094-supplementary.pdf]

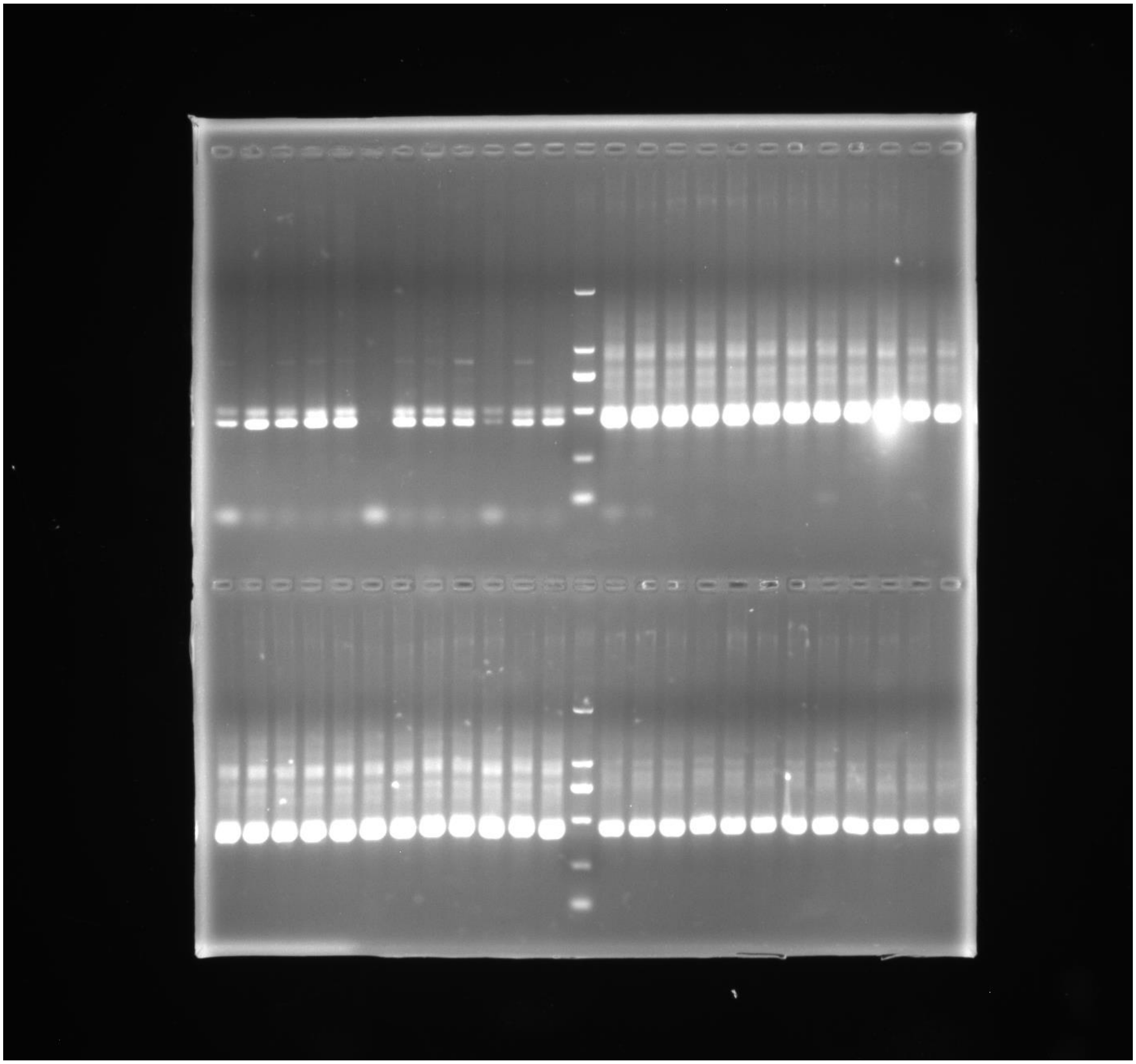

Figure S1. Uncropped PCR amplification results for *empA* gene fragment (439 bp) from *V. anguillarum* isolated from infected fish samples.

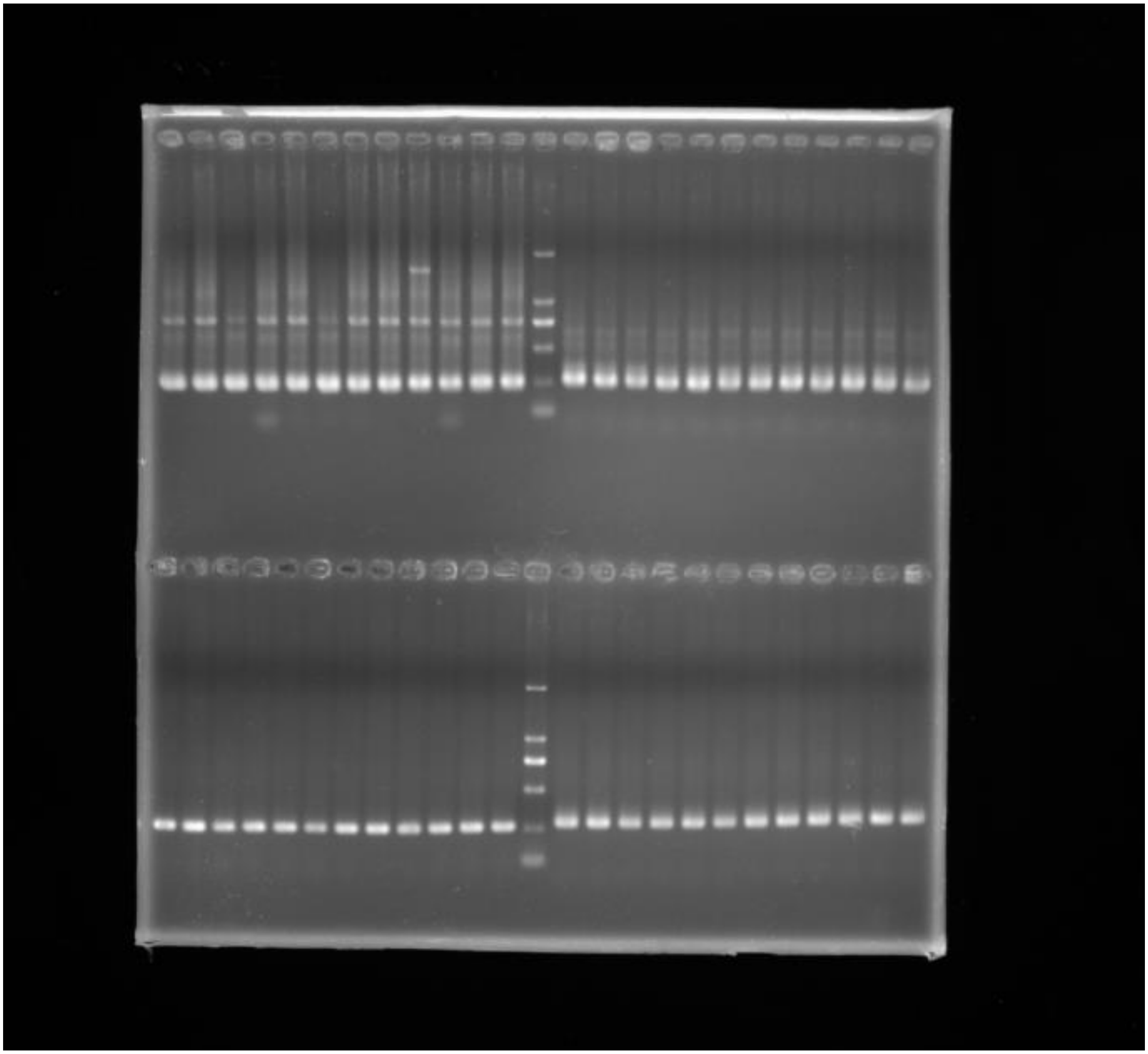

Figure S2. Uncropped PCR amplification results for *Sin* gene fragment (300 bp) from *S. iniae* isolated from infected fish samples.

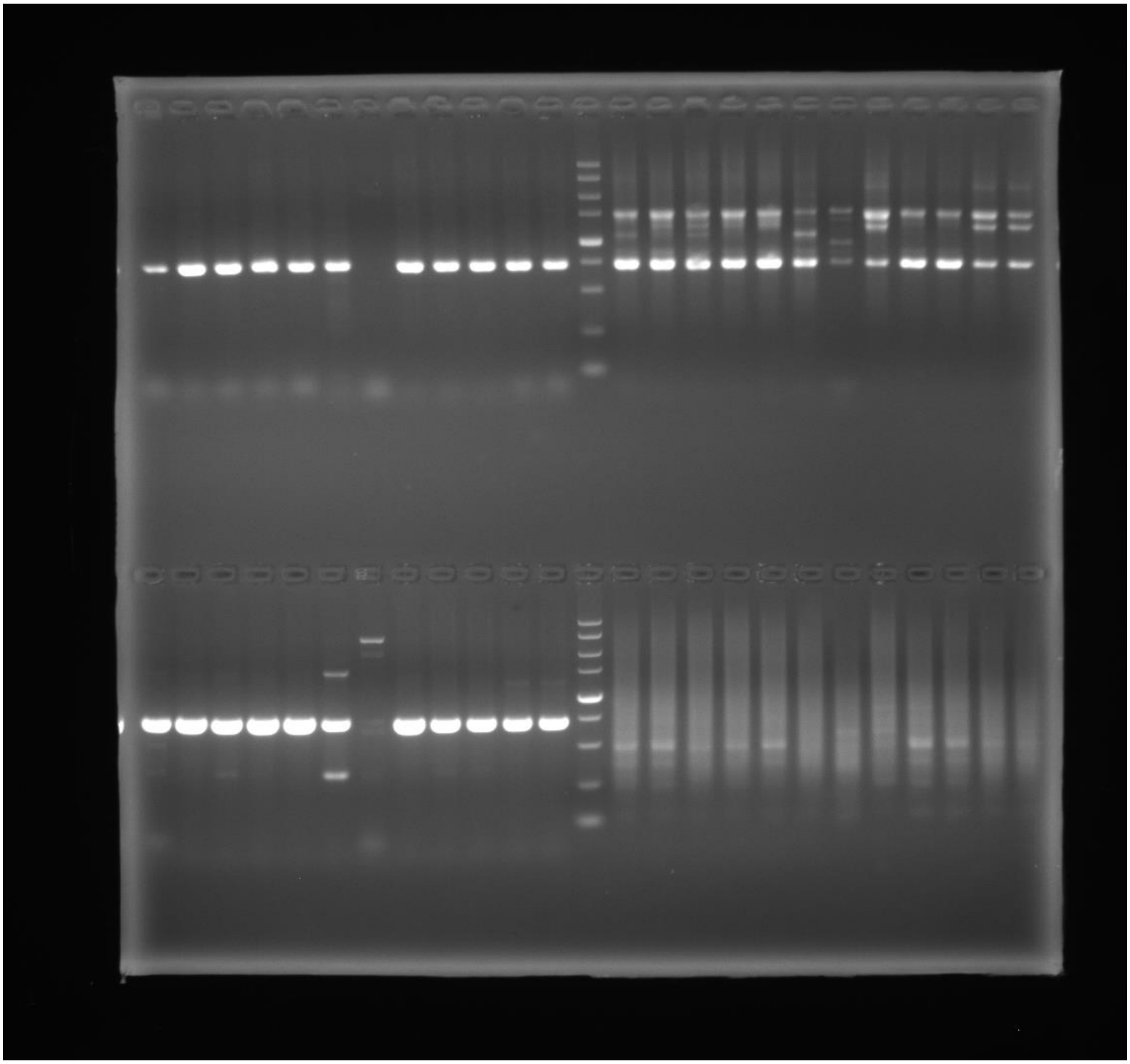

Figure S3. Uncropped PCR amplification results for *empA* gene fragment (439 bp) from *V. anguillarum* isolated from infected fish samples.
